# Familiarity and Use of Veterinary Services by US Resident Dog and Cat Owners

**DOI:** 10.3390/ani10030483

**Published:** 2020-03-13

**Authors:** Courtney Bir, Mario Ortez, Nicole J. Olynk Widmar, Christopher A. Wolf, Charlotte Hansen, Frederic B. Ouedraogo

**Affiliations:** 1 Department of Agricultural Economics, Oklahoma State University, Stillwater, OK 74078, USA; 2 Department of Agricultural Economics, Purdue University, West Lafayette, IN 47907, USA; mortez@purdue.edu (M.O.); nwidmar@purdue.edu (N.J.O.W.); 3 Charles H. Dyson School of Applied Economics and Management, Cornell University, Ithaca, NY 14853, USA; caw364@cornell.edu; 4 American Veterinary Medical Association, Veterinary Economics Division, Schaumburg, IL 60173, USA; CHansen@avma.org (C.H.); FOuedraogo@avma.org (F.B.O.)

**Keywords:** consumer behavior, pet care, procurement of veterinary services, veterinary medicine, veterinary services

## Abstract

**Simple Summary:**

Demographic information including pet ownership, veterinary use, and beliefs regarding veterinary care were collected from 997 U.S. residents. Approximately half of respondents had a dog, or had a dog in the past five years, while 37% of respondents had a cat. Veterinary visits differed between cat and dog owners, with over 90% of dog owners visiting a veterinarian at any time and 40% of cat owners visiting a veterinarian at any time. Using logit models, the likelihood of visiting a veterinarian increased with the age and income of the pet owner. Being a cat owner decreased the likelihood of visiting the veterinarian.

**Abstract:**

Pet ownership, veterinary use, and beliefs regarding veterinary care were elicited through the use of a nationally representative survey of 997 U.S. residents. Fifty-one percent of respondents have or had a dog in the past five years and 37% have or had a cat in the past five years. Over ninety percent of cat and dog owners had visited a veterinarian at any time, but only about 40% visited a veterinarian annually. With the rise of options in veterinary medicine, including low-cost options for vaccines and spay/neuter, further study and analysis of pet-owners use of veterinary care is warranted. Fifty-four percent of dog owners and 40% of cat owners who went to a low-cost spay/neuter clinic also went to a veterinarian/clinic/practice. This finding suggests that pet-owners who use low-cost options do so in a manner that supplements rather than replaces traditional veterinary care. Logit models were employed to evaluate the relationship between dog and cat owner demographics and visiting a veterinarian. The probability of visiting a veterinarian increased with age and income for dog owners.

## 1. Introduction

Over the past thirty years there has been a rise in the number of households with pets, which now hovers at around 57 percent (including dogs, cats, birds, horses, and exotic/specialty pets) [1]. At the end of the year 2011 the number of dogs was estimated at 70 million and the number of cats was estimated at 74.1 million, and in 2018 the number of dogs was estimated at 89.7 million and the number of cats was estimated at 94.2 million [2]. Given the growth in pet populations, there is a clear need for animal care services [1]. Basic animal needs are often characterized by the five freedoms: freedom from hunger and thirst; freedom from discomfort; freedom from pain, injury, and disease; freedom to express normal behavior; and freedom from fear and distress [3]. Veterinary services help pet owners meet the basic level of care outlined by the five freedoms, but not all pet owners meet the veterinary needs of their pet, for one reason or another [2]. According to the 2017–2018 American Veterinary Medical Association (AVMA) pet ownership and demographic sourcebook, while 67% of dog owners and 41% of cat owners received veterinary care from a clinic, hospital, or house call, the remainder sought services from other providers, with the exception of 21% of dogs and 52% of cats not obtaining any routine or preventative care [1]. This equates to twenty-seven percent of all pets not being seen or examined by a veterinarian in 2016 [1]. Understanding the growing pet owning population’s preferences for the use of veterinary care is of importance to the veterinary community, and may change over time, warranting frequent updates in information, made easily accessible. 

The concept of One Health makes the health of animals, including pets, important for all people [4]. “CDC’s One Health Office recognizes that the health of people is connected to the health of animals and our shared environment. A One Health approach encourages collaborative efforts of many experts (like disease detectives, laboratorians, physicians, and veterinarians) working across human, animal, and environmental health to improve the health of people and animals, including pets, livestock, and wildlife” [4]. Veterinarians serve as an important contributor to animal health, and the understanding of veterinary services and perceptions of veterinarians by animal owners is important to recognizing potential pitfalls in the animal–human health symbiosis. 

The current pet service and veterinary care marketplace includes the traditional veterinary hospital or clinic, which may include house calls, and has extended to include mobile clinics, animal shelters, and humane societies, county, city, or other public sponsored events, and pet superstores or pet shops. Additionally, recent expansions in pet services and products, including online pet pharmacies [5], online food and product delivery (including via subscription services) [6], and non-traditional veterinary medical service offerings (e.g., vaccine clinics provided outside of veterinary clinics or offering extended hours) necessitate revisiting consumer expenditure and buying behavior regarding pet products. The dynamic nature of the pet services and product industry provides pet owners with ever-changing options for their pet care, meaning veterinarians are often presented with new competition and changing consumer expectations. The extent to which pet owners are utilizing new options to augment or replace traditional veterinary clinic care are not currently understood.

This analysis characterizes pet ownership and the subsequent care of the pet. The sample was not targeted at pet owners specifically, but instead was targeted to be representative of the U.S. in terms of key census demographic categories, which allowed for a natural proportion of the sample to self-identify as pet owners. Therefore, characteristics of the sample, including general pet care, were elicited to characterize the sample. More specifically, the rates of usage and preferences for veterinary medical services and service providers by pet owning households were elicited. In-depth knowledge of, familiarity with, and use of veterinary procedures is reported. The relationship between demographics and the use of veterinary care for both dog and cat owners were established using logit models. More broadly, this analysis investigates the use of non-traditional and low-cost veterinary medical services to determine how, and by whom, such services were used. The mix of veterinary services employed, as well as the reasoning behind the decision to seek or not seek veterinary care in that manner were evaluated. Fundamentally, this analysis seeks to determine if low-cost services are used alongside other more traditional veterinary clinics or utilized primarily by households in place of traditional veterinary services. This information can be used by those in the veterinary industry to help target areas that are not meeting consumers’ needs, and to anticipate changes in usage of veterinary services. 

## 2. Materials and Methods 

An online survey instrument, which was approved by the university Institutional Review Board (IRB), was designed to elicit information related to public perceptions and self-reported experience with veterinary medical services. The survey was administered from July 9, 2019 to July 18, 2019 using Qualtrics, an online survey tool, to accumulate household demographic information, pet-related spending behaviors (with explicit focus on dog and cat product and service related spending), and self-reported familiarity with veterinary medical practices and procedures by U.S. residents. A company that hosts a large opt-in panel database, Kantar, was used to obtain survey respondents. Respondents were required to be 18 years of age or older to participate. Using quotas, the sample was targeted to be representative of the U.S. population in terms of gender, income, education, and geographical region of residence [7]. Regions of residence were defined as in the Census Bureau Regions and Divisions.

To understand the composition of pet owning households, respondents were asked if they had pets in the past five years, or if they were planning to acquire a pet in the next five years. Respondents who indicated they currently had a pet, or had one in the past five years, were asked to indicate what kind of pet they had. If the respondent indicated they currently had a cat or dog, or had one in the past five years, they were asked a series of additional questions including whether they were the primary caregiver, the type of care provided (including veterinary care), and expenditures on veterinary products and services. There are some trade-offs associated with including respondents who had a pet in the past five years, as opposed to only current owners, in the analysis. After the loss of a pet, people may take days, weeks, or even years to get a new pet [8]. Additionally, elderly people may not replace deceased pets, and both the young or old may have to temporarily or permanently rehome pets as living situations change. However, the behavior and beliefs regarding veterinary medicine of these groups are important for those in the veterinary medicine industry, although the groups themselves may be different. It can be noted that as time passes, respondent recall of past expenditures may not be as accurate. In addition, although inflation has been low the past 5 years, there may be some inflation impact on reported spending of respondents who had a pet in the past. Nonetheless, both current and past pet owners were included in the analysis as both groups have experience caring for pet animals, including making decisions about their medical care.

Understanding respondents’ preferences for, and experience with, dog and cat veterinary products and services was an important component of this data collection effort. Self-reported familiarity and/or experience with various veterinary services, ranging from common practices such as annual exams or spay/neuter surgeries to rarer services such as amputation and/or anti-anxiety medications for pets was collected. Familiarity with veterinary services was compared across household demographics as well as between dog and cat owners. The use of veterinary care, including how their veterinarian was chosen, preferences regarding their veterinarian, and the type of clinic frequented were also elicited from respondents. 

Frequencies were calculated for all categorical variables and means were calculated for continuous variables. The test of proportions was conducted to determine the statistical representativeness of the survey respondents by comparing the percentage of the targeted demographic groups from the sample to the U.S. census. In addition, the test of proportions was employed to study statistical differences in rates of use and/or familiarity among owners of dogs versus cats, as appropriate.

Logit models were estimated in STATA 15 to identify the relationship between currently or previously owning a cat or dog and seeking veterinary care (Appendix A). Logit models were chosen because the probability of visiting the veterinarian takes the form of either 0 or 1, meaning the respondent either did or did not visit the veterinarian [9]. Two independent logit models were estimated, one for taking a dog to the veterinarian and one for taking a cat to the veterinarian. It is important to note that some respondents had both cats and dogs and therefore would be present in both samples. The latent utility associated with seeking a veterinarian (*SeekVet_i_*) for respondent *i* can be represented by the equation [9]:

(1) $$SeekVet_{i}=\beta_{1}Male_{i}+\beta_{2}Age_{i}+\beta_{3}Income_{i}+e_{i}$$

More specifically, the dependent variable took the value 1 for respondents who said they seek veterinary care at least once a year, based on the question “How often do (did) you seek veterinary care for your dog(s)/cat(s)?”, and 0 for respondents who indicated “Never”, “Only in emergencies”, or “I do not know”. Male took the value of 1 if the respondent was male, and 0 if the respondents was female. The independent variables age and income were incorporated as categorical variables. Age remained in the categories as defined in Table 1 and was assigned the numbers 1 through 6 in ascending order. Income remained in the categories as defined in Table 2 and were assigned numbers 1 through 5. The unobserved error term is represented *e*. If the error term is assumed independently, identically, distributed extreme value, the logit probability (*P_i_*), for respondent *i* becomes:

(2) $$P_{i}=\frac{e^{\beta^{\prime }x_{i}}}{\sum e^{\beta^{\prime }x_{i}}}$$

where *x_i_* is the vector of observed variables as outlined in Equation (1). For ease of interpretation, marginal effects were calculated [9,10].

## 3. Results

The total number of survey respondents was 997; demographics of the respondents closely matched the targeted demographics of the U.S. population as documented by the U.S. census with few statistical differences (Table 1). There was a lower percentage of respondents in the sample who were aged 18–24 years (8%), did not graduate from high school (4%), and from the Midwest (21%) when compared to the U.S. census: 13%, 13%, and 38%, respectively. There were higher percentages of respondents who attended college, Associate’s or Bachelor’s degree earned (32%) and from the South (38%) when compared to the U.S. census: 27% and 21%, respectively. 

Sixty-three percent of respondents indicated that they currently own a pet; nine percent do not currently have a pet but had one in the past five years, and eight percent said they plan to acquire a pet in the next five years (Table 2). The demographics of those who indicated they currently have a pet were compared to that of non-pet owners (Table 1). The percentage of pet owners was statistically higher than non-pet owners in the following categories: aged 25–34 (21% pet owners, 15% non), and aged 35–44 years (20% pet owners, 14% non). The percentage of pet owners was statistically lower than non-pet owners in the following categories: male (44% pet owners, 57% non), aged 65 years and older (14% pet owners, 29%, non), and having an income of $0–$24,999 (20% pet owners, 27% non). The species and number of pets currently owned (n = 629) and previously owned (n = 94) are presented in Table 2. Some respondents owned all species considered at least; however, cats and dogs were by far the most prevalent pet species.

Respondents who indicated they currently own or owned a dog (n = 505) or a cat (n = 367) over the past five years were asked questions regarding past veterinary care usage, and their answers were compared using the test of proportions between dog and cat owners (Table 3). High percentages of both dog and cat owners indicated they were the primary caregiver of the cat (88%) or dog (90%). For both dogs and cats, common acquisition methods selected by pet owners were purchased, or adopted/rescued. A higher percentage of dogs (40%) were purchased when compared to cats (17%). Conversely, a lower percentage of dogs (55%) were adopted, compared to 72% of cats. Nearly half of both dog (49%) and cat (44%) owners indicated they had an annual veterinary visit for preventative health. For cat owners, 36% of respondents indicated they did not participate in any of the actions presented, which was higher than the percentage of dog owners, 16%. A higher percentage of dog owning respondents regularly exercised or walked their dog (38%) when compared to cats (12%). Statistically significant differences were not found between the percentage of cat and dog owners who subscribe to or follow veterinary health experts or sources on social media (9% for both cat and dog owners), or visiting with a behavioral specialist (8% for both cat and dog owners). Sixteen percent of dog owners had participated in a formal obedience class for their dog. 

Over 40% of dog and cat owners indicated they sought veterinary care once a year (Table 3). A statistically higher percentage of cat owners indicated they never seek veterinary care (7%) or only seek veterinary care in emergencies (28%), when compared to the percentage of dog owners, 2% and 15% respectively. A higher percentage of dog owning respondents (35%) indicated they took their dog to the veterinarian more than once a year when compared to the percentage of cat owners (20%). A higher percentage of dog owners (69%) indicated they visited a veterinarian/clinic practice of any kind when compared to cat owners (59%). Additionally, a higher percentage of dog owners visited an emergency veterinary clinic (24%), or a veterinary surgery center (9%) when compared to cat owners, 15% and 4% respectively. A higher percentage of cat owners (39%) used a low-cost spay/neuter clinic when compared to dog owners (32%). The same percentage of dog and cat owners used a low-cost vaccination clinic (28%), and veterinary college provided services (4%). Statistically significant differences were not found between the percentage of dog and cat owners who used ambulatory veterinary services (6% dog owners, 5% cat owners), and specialty veterinary service center/clinic (5% dog owners, 4% cat owners). To better understand the mix of types of veterinary clinics dog and cat owners were utilizing, the overlap in use was broken down for dog owners in Table 4 and cat owners in Table 5. Fifty-four percent of dog owners and 40% of cat owners who went to a low-cost spay/neuter clinic also went to a veterinarian/clinic/practice of any kind. For those respondents who went to a low-cost vaccination clinic, 56% of dog owners and 43% of cat owners also went to a veterinarian/clinic/practice of any kind. 

Over 40% of dog and cat owners indicated that cost prevented them from seeking veterinary care. However, statistical differences between cat and dog owners were not found (Table 3). Twenty-six percent of both dog and cat owners indicated they did not seek veterinary care because their pet did not get sick or injured. Nineteen percent of dog owners and 18% of cat owners selected convenience as an item that prevented them from seeking veterinary care. Low percentages of respondents (2% for both cats and dogs) selected unreliable transportation as a preventative item. Over 50% of dog owners and cat owners indicated that discounts would or would have incentivized them to seek veterinary care. Dog and cat owners (10% of respondents) selected telemedicine least frequently as a method that would incentivize seeking veterinary care. 

Out of 505 dog owners and 367 cat owners, 95% (n = 480) and 91% (n = 332) indicated that they seek veterinary care for their pets, respectively. Respondents who sought veterinary care were asked additional questions regarding their views and use of veterinary services (Table 6). Location (62% of dog owners and 60% of cat owners) and quality of care (52% of dog owners and 49% of cat owners) were the main reasons chosen by respondents for selecting a specific veterinarian or clinic. Respondents also frequently selected the veterinarian’s knowledge (41% of dog owners, and 34% of cat owners) and pricing (38% of dog owners and 42% of cat owners) as a reason for choosing that veterinarian/clinic. Although pricing was selected by high percentages of respondents, only 15% of dog and 13% of cat owners selected payment plans. A higher percentage of dog owners selected convenience of hours as an important reason (23%) when compared to cat owners (17%). Low percentages of dog owners (5%) and cat owners (4%) selected multi-pet discounts as a reason for selecting their regular veterinarian/clinic.

Regarding preferences for specific veterinarians within a clinic/practice, high percentages of veterinarian visiting respondents indicated they have no preference among veterinarians (39% dog owners and 42% cat owners) or have some preference but will see other veterinarians if unavailable or inconvenient scheduling (38% dog owners and 38% cat owners) (Table 6). By far most respondents classified their veterinarian as a local independent clinic/veterinarian (80% dogs and 70% cat owners). However, 48% of dog owners and 43% of cat owners reported the veterinary clinic they most often frequent is associated with Banfield, Veterinary Centers of America (VCA), BluePearl, or Pet Partners.

Respondents who currently own or have owned a dog (n = 505) or a cat (n = 367) were asked about their familiarity with different veterinary procedures (Table 7). High percentages of respondents had personal experience with vaccinations (66% of dog owners, 60% of cat owners), wellness exams (56% of dog owners, 52% of cat owners), flea and tick preventatives (60% of dog owners, 56% of cat owners), and spaying and neutering (56% of dog owners, 58% of cats). Higher percentages of dog owners had experience with heartworm tests (49%), heartworm prevention (54%), and pain relievers through either prescriptions or over-the-counter means (32%), when compared to cat owners, 35%, 42%, and 26% respectively. More than 40% of cat and dog owners were not familiar with pre-anesthesia bloodwork, chemotherapy, and/or radiation, and chiropody/acupuncture.

Both cat and dog owners spent money on a range of veterinary related products including veterinary care, prescription items, and other services such as boarding and grooming specifically at the veterinary clinic (Table 8). Note that unless specifically stated, the product could have been purchased from the veterinarian, or through another company/store. Veterinary care included services provided by the veterinarian that did not include food or medication. These goods were listed separately as they can be procured in places other than the veterinarian’s office, such as online with a valid prescription. In general, higher percentages of dog owners spent greater than $75 per year per dog on veterinary care. Seventy-eight percent of dog owners indicated they spend less than $225/year on veterinary care compared to 86% of cat owners. Ten percent of dog owners and 23% of cat owners indicated they do not spend any money on veterinary care for their pets. The majority of cat and dog owners (72% dog owners, 74% cat owners) did not purchase prescription food (Table 8). Forty-seven percent of dog owners and 41% of cat owners indicated they spent between a penny and $75 dollars on flea and tick preventatives. Fifty-four percent of cat owners and 27% of dog owners did not purchase heartworm preventatives. High percentages of both cat and dog owners did not board or groom their pet at the veterinarian.

Logit models were estimated in order to study the impact of demographics on the likelihood to regularly seek veterinary care for either their dog or their cat (Table 9 and Table 10). The cat model was not statistically significant; however, the dog model was. Being male did not have a statistically significant effect on taking a dog to the veterinarian. As age increased and as income increased, the probability of taking a dog to the veterinarian increased. 

## 4. Discussion

The specific breakdown of pet ownership across U.S. households is not well documented, and changes over time. Thus, targeting the sample to be representative of the U.S. population, whether the respondent was a pet owner or not, was the approach used. The sample analyzed in this manuscript closely mirrored the U.S. population; however, it was slightly over-educated. Over-education is common in online surveys [11]. However, the benefits of online surveys including short completion time, and affordable implementation are well accepted [12,13].

The majority of both cat and dog owners indicated they were the primary caregiver of their pets. It seems unlikely that nearly all the pet owners in the study were the primary caregiver, given pet owners, and more specifically primary care-givers were not targeted. However, these respondents self-reported themselves as primary caregivers, a belief or perception which is expected to impact on an individual’s views towards animal care. For both dog and cat owners, common acquisition methods selected by pet owners were purchased, or adopted/rescued. Similar to these findings, in their representative sample of U.S. residents, Bir et al. [14] found that 37% of dog owners had purchased a dog, and 40% of dog owners had adopted a dog. In a best–worst scaling experiment of the most ethical ways to acquire a dog, Bir et al. [15] found that adoption had by far the largest preference share (79%) when compared to other methods including purchasing, finding a stray, gift, or others. Despite considering adoption being one of the most ethical ways to acquire a dog, many people choose to purchase a dog. Dogs serve various purposes, including companionship, status symbols such as the Victorian era lap dogs, and working dogs including the prestigious Russian Rottweiler guard dogs of the 1990s [16]. Ghirlanda et al. [17] found that popularity spikes in different dog breeds were not correlated with breed characteristics but were more likely a function of fashion. Maddalena et al. [18] found that common reasons for not wanting a shelter dog included wanting a purebred dog and being uncertain that a shelter could provide the type of dog they desired, which echoes the importance of breed and function to dog owners. Adoption, as a popular method of acquisition, is an important note for the veterinary industry. Some larger veterinary chains such as Banfield Pet Hospital and VCA have partnered with animal shelters to offer free exams to new adopters [19,20]. Partnering with shelters, or advertising at adoption events may be one way for veterinarians to reach potential veterinary clients.

A higher percentage of dog owning respondents regularly exercised their dog when compared to cat owners. Bir et al. [21] asked the general population and veterinarians their level of agreement on a scale from 1 to 7, and veterinarians had a statistically higher level of agreement that cats needed exercise when compared to the general population. Perception of the rigor of animals’ exercise may be one explanation for the differences in dog versus cat exercise. An alternative hypothesis explaining the possibility of underreporting cat exercise is that since many forms of cat exercise, such as using a laser pointer, do not require exertion of the owner (as walking a dog would) they are less memorable as exercise events. Obesity is a growing problem in both pet cats and dogs, which can cause health issues ranging from orthopedic disease to diabetes [22]. It is recommended that both cats and dogs have a dietary plan focused on weight management, and that exercise be increased along with behavioral management to decrease obesity [22].

Visiting with a behavioral specialist was reported by 8% of both cat and dog owners. Although a behavioral specialist can help identify and asses the relationship between a patient’s health and behavior [23], it was somewhat surprising that higher numbers of dog owners did not seek this type of care given the general size and management differences of the species. Dog bites account for 85% to 90% of animal bites (still only 1% of injury related emergency department visits) in the U.S. while cat bites account for only 5% to 10% [24]. However, 16% of dog owners had participated in an obedience class, which may replace the need for a visit to the animal behaviorist. Animal behavior as a specialty is relatively new, gaining full recognition in 1993 [23]. As the number of behavioral specialists increase, and the consultation of such specialists become more commonplace, future studies will be needed to further evaluate the usage and impact of this newer veterinary service. 

Forty-six percent of dog owners and 44% of cat owners had an annual veterinary visit for preventative health. In a targeted survey of pet owners, the AVMA found that 79% of dog owners and 48% of cat owners sought routine veterinary care for their pet [1]. The difference in numbers could be due to the interpretation of the word routine, which for some people may not include annual checkups despite veterinary recommendations. Additionally, people may feel pressure to indicate they have visited a veterinarian when responding to an AVMA survey, as opposed to an academic survey. In this survey, high percentages of respondents selected veterinary services spending category $0.01–$75 for cats and $76–$150 for dogs. According to the AVMA survey, the average expenditure per household at the veterinary clinic was $373 per year, $161 per visit, and $27.7 million in total in 2016 [1]. This survey allowed for a further breakdown of potential veterinary services such as food and medication. Prescription food and medication are being made available online directly through the manufacturer or through third party sites such as 1-800-PetMeds and Chewy [25,26]. The veterinary industry needs to be aware of spending on goods and services that could potentially leave the clinic setting, as this may lead to decreases in revenue.

Likely due to their smaller size and lower probability of harming humans and large livestock, in general, U.S. laws are lax regarding cat licensing and containment, which has contributed to a population of free-roaming cats [27]. Neighborhood or community cats may be feral, or semi-feral, and although they have some form of human care, they are not considered the same as a household pet cat. Trap–neuter–return (often referred to as TNR) programs have become a popular way to begin to control the feral cat population, without the extermination of existing cats [28]. Additionally, “barn cats”, a term which applies to any cat that helps keep a barn area vermin-free, are often semi-feral, non-social, or feral [29]. It is recommended that these cats still get veterinary exams, vaccines, and be spayed/neutered; however, it is often difficult to get such cats to a traditional veterinarian [29]. The higher percentages of cat owners, when compared to dog owners, who participate in low-cost spay/neutering may in part be driven by cats who are not typical house cats, including neighborhood cats and barn cats. In general, low-cost options including both spay/neutering and vaccination are used in conjunction with other types of veterinary care. Future research could determine under what circumstances respondents choose to visit low-cost options. It is unclear if pet owners are choosing low-cost options for certain pets under specific circumstances, or for some stages of the pet’s life only. It has been previously documented that pet owners who sought alternative service providers to a stand-alone clinic/hospital/house calls, paid less per visit [1]. What is clear is that most respondents are not using low-cost options to completely replace traditional veterinary services.

Considering reasons for selecting their regular veterinarian/clinic, location was selected by high percentages of both dog and cat owners. Neill et al. [30] considered the impact of clinic location on veterinary income. As the density of veterinary clinics increased, income decreased, and income per capita did not impact veterinary income [30]. In this study, few respondents selected multi-pet discounts as a reason for selecting their regular veterinarian. This was perhaps reflective of the pet composition of most of the households. Fifty-one percent of respondents only had one dog and 34% of respondents only had one cat. Unsurprisingly, price/cost was selected by a high percentage of respondents as an item that prevented veterinary care. Previous literature did not focus on the reasons why pet owners chose their veterinarian in the same manner as this study. Given the changing landscape of veterinary medicine, future work should continue to evaluate why pet owners are choosing their veterinarian or type of clinic specifically. Although high percentages of respondents (over 50% of cat and dog owners) indicated that discounts would have incentivized them to seek veterinary care, very low percentages of respondents indicated that the availability of payment plans were a reason for selecting their regular veterinarian or clinic. According to the AVMA survey, lower-income households spend less at the veterinarian than higher-income households [1]. Discounts may not be the solution to increasing veterinary care. National pet dental health month, which occurs in February, was originally created to promote oral health care for pets [31]. The month of February has morphed into a dental discount month for many clinics, where dental cleanings are discounted if done in February [32]. This has resulted in many clients delaying needed care until February, or simply scheduling annual cleanings for that month in particular, which may result in the client believing the cost is too high, or that dentals are not medically necessary [32]. Although discounts may serve to increase demand for veterinary services in the short term, if prices are set to reflect clinic expenditures, steep discounts cannot be a regular business practice for a clinic to remain profitable. 

Despite the majority of respondents indicating they went to a local independent clinic/veterinarian, nearly half of respondents indicated they went to a franchised or “chain store” veterinary clinic including Banfield, VCA, BluePearl, or Pet Partners. These two things cannot be simultaneously true; therefore, it is likely respondents believed their veterinary clinic was independently-owned. Looking at the overarching company websites, wording was carefully chosen such as local [33], neighborhood [33,34], and community [35] to describe the parent companies which have locations available across the U.S.A. It is likely respondents have greater concern about the location of the physical clinic they are visiting, and simply care less about the prevalence of the parent company. Despite the likely confusion regarding the classification of the veterinary clinic, the popularity of these chain options indicate that they are providing a level of convenience, preferred location, consistency, or familiarity that pet owners want. Corporately owned veterinary clinics began 30 years ago when VCA acquired their first veterinary clinic in 1987. Currently, it is estimated that about 10% of companion animal practices and 40%–50% of referral practices are owned by corporations [36]. Corporate veterinary clinics attract veterinarians by offering signing bonuses and assistance with veterinary school debt [36].

Although many pet owning respondents were personally familiar with veterinary practices that necessitate anesthesia including dental cleaning/tooth extractions, and spay/neuter, more than 40% of cat and dog owners were not familiar with pre-anesthesia bloodwork. The American Animal Hospital Association (AAHA) guidelines recommend that all senior pets have pre-anesthesia blood work done, if not done in the previous six months [37]. Many individual clinics recommend pre-anesthesia blood work prior to all surgeries, and a quick Google search shows a plethora of pre-anesthesia opt-out forms that state the risks associated with anesthesia. It is possible that respondents simply forgot to talk to their veterinarian about pre-anesthesia blood work, or that a more detailed conversation by veterinarians and staff is necessary to convey the importance of such testing to clients.

Although historically it was believed that animals did not feel pain in the same manner as humans, today we have a better understanding of pain and pain management in animals, which is similar to humans [38]. Despite cat and dog owners having high rates of personal familiarity with surgical procedures including spay/neutering and dentals, which would likely require pain management, cat owners had less familiarity with pain management. Additionally, lower percentages of cat owners used either prescription or over the counter pain relievers. Procedures and conditions that cause pain in dogs are routinely overlooked in cats, although cats should have the same pain management care [38]. Bir et al. [21] found that on a 7-point scale, the general population had higher agreement than veterinarians that cats tolerate pain well, and veterinarians agreed more that it is important to alleviate pain in cats.

Similarly to the 54% of respondents who used heartworm prevention in this study, Bir et al. [14] found that 55% of dog owners used heartworm prevention continuously in their nationally representative study of U.S. residents. Other studies have found heartworm preventative use compliance of less than 50% [39,40]. The American Heartworm society recommends that all pets be on continuous heartworm preventative and warns that no U.S. state is heartworm free [41]. Dogs are a natural host for heartworms, and the heartworm inside a dog can mature to an adult, mate, and reproduce [42]. Cats are an atypical host for heartworms, and the heartworm cannot mature inside the cat [42]. However, cats can still suffer from heartworm associated respiratory disease, and the treatments available in dogs cannot be used in cats [42]. Therefore, the only treatment for heartworms in cats is prevention [42]. Despite the recommendations for cats to be on heartworm medication, and the lack of treatment options, lower percentages of cat owners had experience with heartworm tests and prevention when compared to dog owners in this study. It is possible that cat owners do not understand the risks associated with heartworms, or do not see the benefit of heartworm preventative in cats since the disease is not as dramatic or prevalent as it is in dogs. 

In the logit model of pet owner veterinary clinic attendance, the probability of visiting a veterinarian increased with increased age and higher income in the dog model. The logit model for cat veterinary attendance was not statistically significant. Lower-income household dog owners who were younger were the most vulnerable for not getting the recommended veterinary care for their pet(s). Focusing on the amount of time, in hours, spent at the veterinarian, Neill et al. [43] found that male pet owners and older pet owners spent less time at the veterinarian. In a study of pet owner expenditure, Henderson [44] found that financial issues were a barrier for pet owners when it came to preventive, sick, and emergency care. Although the model for cat veterinary clinic attendance was not statistically significant, in general AVMA [1] found that being a cat owner decreased the probability of seeking veterinary care. According to the pet owner’s economic value study, dog owners are also willing to spend more than cat owners for veterinary services [45]. As seen in in the AVMA’s Pet Ownership and Demographics Sourcebook, more dog owners utilized other veterinary service providers (that were not clinics, hospitals, and house calls), than cat owners [1]. Simply stated, pet owners spent more on their dogs than cats, and took dogs to the vet more often than cats [1]. It is unclear from this work what characteristics drive cat owners to visit the veterinarian. There may not be a systematic reason why some cat owners visit the veterinarian at least once a year and others do not. Although demographics did not shed light on cat veterinary visits, further studies of cat owners specifically could delve deeper into cat owner preferences for veterinary care. Considering the popularity of cats as pets in the U.S., creating a stronger culture of veterinary care for cats could benefit feline health as well as the veterinary industry.

## 5. Conclusions

The most commonly owned pets in the nationally representative sample were cats and dogs. Veterinary service use differed between cat and dog owners, including the rate of visitation, the frequency, and type of care sought. Nearly half of the dog and cat owners visited the veterinarian once a year, with over 90% visiting a veterinarian at least sometimes. Veterinary care options are expanding, with offerings beyond the traditional brick and mortar independently-owned veterinary clinics. Cost remains a concern for pet owners; over 40% of dog and cat owners indicated that cost prevented them from seeking veterinary care. Some pet owners have turned to low-cost spay/neutering and vaccination clinics for care. The usage of low-cost providers or single purpose clinics, such as spay/neutering clinics or vaccine drop-in clinics has been a point of conversation within veterinary medicine. Understanding whether dog and cat owners are using low-cost providers as a complement to traditional veterinary medical care or as a replacement to a primary veterinarian can aid decision making within the profession. Despite using such clinics, high percentages of both cat and dog owners still visited traditional veterinary clinics in the sample of respondents studied in this analysis. This evidence suggests that pet owners are using these low-cost services under certain circumstances to augment regular veterinary care. The exact scenarios, and frequency within a pet’s life that pet owners utilize the low-cost options could be expanded on in future research.

Determining relationships between cat veterinary attendance and demographics remains elusive. Both income and age increased the probability of visiting the veterinarian for dog owners. Understanding why cats do not frequent the veterinarian as often can have important impacts for One Health. Cats are often free-roaming and may be feral or communal cats with many potential contacts with both humans and wild animals. Livestock animals are also an important component of the veterinary industry. This work takes a step towards the better identification of veterinary use and beliefs in two of the most common species of pet animals, but future work is needed to focus on livestock veterinary use. Pet ownership and livestock ownership are fundamentally different, and veterinary care differs widely between species, so different approaches may be needed.

## Figures and Tables

**Table 1:** Survey demographics and demographics of pet owners and comparison between samples, percentage of respondents.

Demographic Variable	Total Respondents (n = 997)	U.S. Census	Currently Owns Pet (n = 629)	Does Not Own Pet (n = 368)
*Gender*				
Male	49	49	44 ^†^	57
*Age*				
18–24	8 ^†^	13	9	7
25–34	19	18	21 ^†^	15
35–44	18	16	20 ^†^	14
45–54	19	17	20	17
55–64	16	17	15	18
65+	19	19	14 ^†^	29
*Income*				
$0–$24,999	22	22	20 ^†^	27
$25,000–$49,999	23	23	23	23
$50,000–$74,999	17	17	18	15
$75,000–$99,999	12	12	13	11
$100,000 and higher	26	26	27	24
*Additional higher income breakdown*				
$100,000–$149,999	15		15	15
$150,000–$199,999	5		5	4
$200,000–$249,999	4		4	2
$250,000–$299,999	1		2	0
$300,000–$349,999	0		0	0
$350,000–$399,999	0		0	0
$400,000+	1		0	1
*Education*				
Did not graduate from high school	4 ^†^	13	4	5
Graduated from high school, Did not attend college	28	28	27	28
Attended College, No Degree earned	21	21	22	21
Attended College, Associates or Bachelor’s Degree earned	32^†^	27	33	31
Attended College, Graduate or Professional Degree earned	14	12	13	15
*Region*				
Northeast	17	18	16	19
South	38 ^†^	21	38	37
Midwest	21 ^†^	38	21	22
West	24	24	24	23

^†^ Percentage of respondents is statistically different than the percentage of the U.S. census or percentage of respondents who have pets and do not have pets is statistically different within demographic categories at the 95% level of confidence.

**Table 2:** Respondent pet ownership including type and number of pets, percentage of respondents.

*Pet animal status percentage of respondents n = 997*							
Currently have pet animal(s)					63		
Do not currently have pet animal(s) but have in the past 5 years					9		
Plan to acquire pet animal(s) in the next 5 years					8		
None					27		
*Number and species of pets respondents currently have, percentage of pet owners n = 629*							
	0	Have at least 1	1	2		3	4 or more
Dog	28	73	51	15		4	3
Cat	45	55	34	13		4	4
Fish	85	15	4	2		2	7
Horse	97	3	1	0		1	1
Bird	92	8	4	2		1	1
Reptile	94	6	3	1		1	0
Rabbit	96	4	2	1		0	1
Small Mammal ^1^	94	5	3	1		0	1
Other	97	3	0	1		1	1
*Number and species of pets respondents have had in the past 5 years, percentage of pet owners n = 94*							
	0	Have at least 1	1	2		3	4 or more
Dog	29	72	49	14		6	2
Cat	68	32	16	9		5	2
Fish	78	22	5	5		4	7
Horse	93	7	5	0		2	0
Bird	90	10	4	2		2	1
Reptile	91	9	6	2		0	0
Rabbit	93	7	2	2		1	2
Small Mammal ^1^	88	12	6	3		1	1
Other	96	4	0	2		1	1

^1^ Small mammals include hamster, ferret, guinea pig, rat, mouse, chinchilla, gerbil.

**Table 3:** Pet owning respondent pet care and usage of veterinary services and statistical comparison between dog and cat owners. Percentage of dog (n = 505) or cat (n = 367) owning respondents.

	Dogs	Cats
*Primary provider of dog/cat care in household*		
Yes	90	88
*How respondents have acquired their dog(s)/cat(s)* ^1^		
Purchased	40 ^θ^	17
Adopted or rescued	55 ^θ^	72
Received as a gift	17	14
Other	4 ^θ^	7
*Action taken regarding dog(s’)/cat(s’) health* ^1^		
Regularly (5–7 x/week) walk or exercise	38 ^θ^	12
Has an annual veterinary visit for preventative health	49	44
Subscribes to or follows on social media veterinary health experts or sources	9	9
Has participated in formal obedience classes with the dog(s)	16	
Has visited with a behavioral specialist	8	8
None of the above	16 ^θ^	36
*Frequency of seeking veterinary care for your dog(s)/cat(s)*		
Never	2 ^θ^	7
Only in emergencies	15 ^θ^	28
Once a year	46	43
More than once a year	35 ^θ^	20
I don’t know	3	3
*Types of practices/service providers used for dog(s)/cat(s)* ^1^		
Low-cost spay/neuter clinic	32 ^θ^	39
Low-cost vaccination clinic	28	28
Veterinarian/Clinic/Practice of any kind	69 ^θ^	59
Emergency veterinary clinic	24 ^θ^	15
Ambulatory veterinary services (i.e., in-home care)	6	5
Veterinary college provided services	4	4
Veterinary surgery center	9 ^θ^	4
Specialty veterinary service center/clinic (e.g., allergy testing, ophthalmologist, etc.)	5	4
Other	3 ^θ^	6
*Items that prevents (prevented) respondent from seeking veterinary care for your dog(s)/cats(s)* ^1^		
Convenience	19	18
Proximity to veterinarian	12	12
Price/cost	40	42
Animal behavior	11	8
Pet stress	9	12
Dog/cat did not get sick or injured	26	26
Unreliable transportation	2	2
None of the above	30	28
*What would (would have) incentivize(d) respondents to seek veterinary care for dog(s)/cat(s)* ^1^		
Discounts	53	54
Mobile care option	24	26
Telemedicine option	10	10
None of the above	32	31

^θ^ Percentage of dog owning respondents is statistically different than the percentage of cat owners at the 95% level of confidence. ^1^ Multiple selections permitted.

**Table 4:** Percentage of respondents who used multiple veterinary service options for their dog, reported number and percentage of respondents who indicated they used each type of veterinary care.

Type of Clinic	Low-Cost Spay/Neuter Clinic (n = 162)	Low-cost Vaccination Clinic (n = 139)	Veterinarian/Clinic/Practice of any Kind (n = 346)	Emergency Veterinary Clinic (n = 120)	Ambulatory Veterinary Services (n = 29)	Veterinary College Provided Services (n = 19)	Veterinary Surgery Center (n = 45)	Specialty Veterinary Service Center/Clinic (n = 27)	Other (n = 15)
Low-cost spay/neuter clinic	162 (100%)	85 (52%)	88 (25%)	33 (28%)	8 (28%)	3 (16%)	15 (33%)	10 (37%)	0 (0%)
Low-cost vaccination clinic	85 (52%)	139 (100%)	78 (23%)	32 (27%)	4 (14%)	4 (21%)	17 (38%)	6 (22%)	1 (7%)
Veterinarian/Clinic/Practice of any kind	88 (54%)	78 (56%)	346 (100%)	90 (75%)	17 (59%)	9 (47%)	33 (73%)	17 (63%)	2 (13%)
Emergency veterinary clinic	33 (20%)	32 (23%)	90 (26%)	120 (100%)	12 (41%)	6 (32%)	18 (40%)	10 (37%)	1 (7%)
Ambulatory veterinary services	8 (5%)	4 (3%)	17 (5%)	12 (10%)	29 (100%)	4 (21%)	7 (16%)	2 (7%)	0 (0%)
Veterinary college provided services	3 (2%)	4 (3%)	9 (3%)	6 (5%)	4 (14%)	19 (100%)	1 (2%)	3 (11%)	0 (0%)
Veterinary surgery center	15 (9%)	17 (12%)	33 (10%)	18 (15%)	7 (24%)	1 (5%)	45 (100%)	6 (22%)	0 (0%)
Specialty veterinary service center/clinic	10 (6%)	6 (4%)	17 (5%)	10 (8%)	2 (7%)	3 (16%)	6 (13%)	27 (100%)	0 (0%)
Other	0 (0%)	1 (1%)	2 (1%)	1 (1%)	0 (0%)	0 (0%)	0 (0%)	0 (0%)	15 (100%)

Note: Shading is provided to indicate that this is a symmetric table.

**Table 5:** Percentage of respondents who used multiple veterinary service options for their cat, reported number and percentage of respondents who indicated they used each type of veterinary care.

Type of Clinic	Low-Cost Spay/Neuter Clinic (n = 144)	Low-Cost Vaccination Clinic (n = 101)	Veterinarian/Clinic/Practice of any Kind (n = 215)	Emergency Veterinary Clinic (n = 56)	Ambulatory Veterinary Services (n = 17)	Veterinary College Provided Services (n = 16)	Veterinary Surgery Center (n = 16)	Specialty Veterinary Service Center/Clinic (n = 13)	Other (n = 23)
Low-cost spay/neuter clinic	144 (100%)	65 (64%)	57 (27%)	21 (38%)	3 (18%)	2 (13%)	6 (38%)	1 (8%)	1 (4%)
Low-cost vaccination clinic	65 (45%)	101 (100%)	43 (20%)	20 (36%)	3 (18%)	8 (50%)	5 (31%)	2 (15%)	0 (0%)
Veterinarian/Clinic/Practice of any kind	57 (40%)	43 (43%)	215 (100%)	40 (71%)	9 (53%)	3 (19%)	6 (38%)	9 (69%)	3 (13%)
Emergency veterinary clinic	21 (15%)	20 (20%)	40 (19%)	56 (100%)	2 (12%)	7 (44%)	9 (56%)	5 (38%)	1 (4%)
Ambulatory veterinary services	3 (2%)	3 (3%)	9 (4%)	2 (4%)	17 (100%)	1 (6%)	2 (13%)	1 (8%)	0 (0%)
Veterinary college provided services	2 (1%)	8 (8%)	3 (1%)	7 (13%)	1 (6%)	16 (100%)	4 (25%)	0 (0%)	0 (0%)
Veterinary surgery center	6 (4%)	5 (5%)	6 (3%)	9 (16%)	2 (12%)	4 (25%)	16 (100%)	1 (8%)	0 (0%)
Specialty veterinary service center/clinic	1 (1%)	2 (2%)	9 (4%)	5 (9%)	1 (6%)	0 (0%)	1 (6%)	13 (100%)	0 (0%)
Other	1 (1%)	0 (0%)	3 (1%)	1 (2%)	0 (0%)	0 (0%)	0 (0%)	0 (0%)	23 (100%)

Note: Shading is provided to indicate that this is a symmetric table.

**Table 6:** Views and use of veterinary services, and statistical comparison between dog and cat owners. Percentage of dog (n = 480) and cat (n = 332) owners who take their animal to the veterinarian.

	Dogs	Cats
*Reasons for selecting regular veterinarian/clinic* ^1^		
Veterinarian’s knowledge	41	34
Quality of care	52	49
Location of clinic	62	60
Pricing	38	42
Availability of payment plans	15	13
Convenience of hours	23 ^θ^	17
Multi-pet discount	5	4
Referred by friend or acquaintance	18	13
Other	2	3
*Preferences expressed for specific veterinarian(s) within the clinic/practice respondent visits*		
Have preference but will see other veterinarians if unavailable or inconvenient scheduling	38	38
Have exclusive preference for a specific veterinarian	23	20
Have no expressed preferences among veterinarians in my clinic/practice	39	42
*Veterinarian Classification*		
Local independent clinic/veterinarian	80 ^θ^	70
Nationally affiliated (chain) clinic/veterinarian	13	15
Mobile pop-up clinic/veterinarian	5	6
Cat only clinic		4
Clinic/veterinarian affiliated with veterinary college	2	4
Other	0 ^θ^	3
*Affiliation of veterinary clinic respondent most often frequents* ^1^		
Banfield Pet Hospitals^®^	14	13
Veterinary Centers of America (VCA)	19	15
BluePearl	7	8
Pet Partners	8	7
None of the above	36	38
I don’t know	30	30

^θ^ Percentage of dog owning respondents is statistically different than the percentage of cat owning respondents at the 95% level of confidence. ^1^ Multiple selections permitted.

**Table 7:** Familiarity with veterinary procedures and statistical comparison between cat and dog owners, percentage of those who have or had a pet (dog n = 505, cat n = 367).

Veterinary Services	I am not Familiar with This Veterinary Service		I am Aware of/Have heard about This Veterinary Service		I Have Personal Experience with This Veterinary Service	
	Dog	Cat	Dog	Cat	Dog	Cat
Pre-anesthesia bloodwork	47	45	31	35	21	20
Fecal test	25	23	39	45	36	32
Heartworm test	14	16	38 ^θ^	49	49 ^θ^	35
Vaccinations	8	10	26	30	66	60
Wellness exam	15	15	30	33	56	52
Heartworm prevention	13	16	32^θ^	42	54 ^θ^	42
Flea and tick prevention	10	10	30	35	60	56
Dental cleaning/tooth extraction	13	13	46	49	40	38
Spay/neuter	13	11	32	31	56	58
Deworming	16	13	49	53	35	35
Pain relievers, prescription/Over the counter (OTC)	25	23	43 ^θ^	52	32 ^θ^	26
Anti-anxiety medications	31	28	51	57	18	15
Surgery under general anesthesia not including spay/neuter	25	22	50	50	25	28
Intravenous fluids (IV)	29	24	45	51	26	26
Chemotherapy and/or radiation	40 ^θ^	33	50	56	10	11
Chiropractory/Acupuncture	49	45	43	47	9	8
Amputation	35	29	55	61	10	10

^θ^ Percentage of dog owning respondents is statistically different than the percentage of cat owning respondents at the 95% level of confidence.

**Table 8:** Annual pet owner spending on veterinary related items per pet, percentage of dog (n = 480) and cat (n = 332) owners.

	Veterinary Care		Prescription Dog Food		Flea/Tick Preventatives		Heartworm Preventatives		Prescription Medications		Boarding at Veterinarian		Grooming at Veterinarian	
	Dogs	Cats	Dogs	Cats	Dogs	Cats	Dogs	Cats	Dogs	Cats	Dogs	Cats	Dogs	Cats
$0	10	23	72	74	15	32	27	54	52	64	69	74	64	74
$0.01–$75	24	29	13	15	47	41	40	33	25	23	15	15	19	15
$76–$150	29	23	7	5	25	16	22	7	12	7	6	4	10	5
$151–$225	15	11	3	3	8	7	6	5	4	2	4	2	2	3
$226–$300	7	5	1	1	4	1	2	1	3	2	2	2	2	1
$301–$375	4	3	1	1	0	1	1	1	2	1	2	1	1	1
$376–$450	2	2	1	0	1	0	1	0	1	0	1	1	1	0
$451–$525	3	1	0	1	0	1	0	0	0	1	0	0	1	0
$526–$600	1	0	0	0	0	0	0	0	0	0	0	0	0	0
$601–$675	1	0	0	0	0	0	0	0	0	0	0	0	0	0
$676–$750	1	1	0	0	0	0	0	0	0	0	0	0	0	0
$751–$825	1	0	0	0	0	0	0	0	0	0	0	0	0	0
$826–$900	0	1	0	0	0	0	0	0	0	0	0	0	0	0
$901–$975	0	0	0	0	0	0	0	0	0	0	0	0	0	0
$976+	2	1	0	0	0	0	0	0	0	0	0	1	1	0

**Table 9:** Logit model results predicting likelihood to seek veterinary care respondents who own or owned a dog (n = 505).

Variable	Coefficient	Standard Error	*p*-Value	Marginal Effect
Male	−0.339	0.240	0.164	−0.050
Age	0.283 ***	0.079	0.000	0.042
Income	0.226 ***	0.079	0.004	0.034
Constant	−0.301	0.387	0.437	
N	505			
Pseudo R2	0.046			
Prob > Chi2	0.000			

Note: *** *p* ≤ 0.001.

**Table 10:** Logit model results predicting likelihood to seek veterinary care respondents who own or owned a cat (n = 367).

Variable	Coefficient	Standard Error	*p*-Value	Marginal Effect
Male	0.086	0.228	0.707	0.020
Age	−0.010	0.073	0.894	−0.002
Income	0.149	0.076	0.049	0.035
Constant	0.110	0.399	0.783	
N	367			
Pseudo R2	0.009			
Prob > Chi2	0.217			

## Appendix A

**Table A1:** Items that prevent (prevented) respondent from seeking veterinary care, by income.

Items	$0–$24,999	$25,000–$49,999	$50,000–$74,999	$75,000–$99,999	$100,000 and Higher
Dogs	n = 90	n = 111	n = 88	n = 69	n = 147
Convenience	11	18	18	22	22
Proximity to veterinarian	9	5	14	20	15
Price/cost	60	43	42	32	29
Animal behavior	9	7	16	16	11
Pet stress	9	4	11	9	10
Dog/cat did not get sick or injured	30	25	25	25	27
Unreliable transportation	4	-	3	1	2
None of the above	19	33	28	29	35
Cats	n = 84	n = 80	n = 69	n = 52	n = 82
Convenience	16	12	15	22	13
Proximity to veterinarian	9	5	10	20	11
Price/cost	42	37	36	32	20
Animal behavior	4	3	6	16	10
Pet stress	9	8	11	9	8
Dog/cat did not get sick or injured	23	22	23	25	14
Unreliable transportation	4	-	-	1	1
None of the above	23	19	24	29	13

**Table A2:** What would (would have) incentivize (d) respondents to seek veterinary care, by income.

Items	$0–$24,999	$25,000–$49,999	$50,000–$74,999	$75,000–$99,999	$100,000 and Higher
Dogs	n = 90	n = 111	n = 88	n = 69	n = 147
Discounts	48	59	57	38	57
Mobile care option	18	13	24	30	33
Telemedicine option	13	5	7	13	13
None of the above	38	35	27	38	25
Cats	n = 84	n = 80	n = 69	n = 52	n = 82
Discounts	54	44	44	25	30
Mobile care option	24	14	19	14	22
Telemedicine option	7	6	5	6	12
None of the above	27	23	22	39	12

**Table A3:** Reasons for selecting regular veterinarian/clinic.

Reasons	$0–$24,999	$25,000–$49,999	$50,000–$74,999	$75,000–$99,999	$100,000 and Higher
**Dogs**	n = 84	n = 106	n = 83	n = 63	n = 144
Veterinarian’s knowledge	27	37	37	49	50
Quality of care	42	44	51	67	55
Location of clinic	60	61	43	57	67
Pricing	50	45	22	29	29
Availability of payment plans	20	8	11	14	13
Convenience of hours	14	23	23	30	22
Multi-pet discount	4	7	5	6	6
Referred by friend or acquaintance	14	24	10	13	17
Other	4	2	2	3	1
**Cats**	n = 73	n = 71	n = 67	n = 41	n = 80
Veterinarian’s knowledge	29	32	33	41	39
Quality of care	45	44	51	56	54
Location of clinic	56	75	54	51	61
Pricing	58	51	37	22	34
Availability of payment plans	23	6	7	12	14
Convenience of hours	21	17	16	17	15
Multi-pet discount	1	3	3	2	8
Referred by friend or acquaintance	10	15	15	12	14
Other	5	1	0	5	4

**Table A4:** Preferences for specific veterinarian(s) within the clinic/practice respondent visits.

Preferences	$0–$24,999	$25,000–$49,999	$50,000–$74,999	$75,000–$99,999	$100,000 and Higher
**Dogs**	n = 84	n = 106	n = 83	n = 63	n = 144
Have preference but will see other veterinarians if unavailable or inconvenient scheduling	44	28	40	33	42
Have exclusive preference for a specific veterinarian	18	23	22	29	25
Have no expressed preferences among veterinarians in my clinic/practice	38	49	39	38	33
**Cats**	n = 73	n = 71	n = 67	n = 41	n = 80
Have preference but will see other veterinarians if unavailable or inconvenient scheduling	33	20	29	25	25
Have exclusive preference for a specific veterinarian	13	10	19	16	13
Have no expressed preferences among veterinarians in my clinic/practice	40	37	33	24	18

**Table A5:** Veterinary Classification, by income.

Veterinarian Classification	$0–$24,999	$25,000–$49,999	$50,000–$74,999	$75,000–$99,999	$100,000 and Higher
**Dogs**	n = 84	n = 106	n = 83	n = 63	n = 144
Local independent clinic/veterinarian	82	88	73	79	76
Nationally affiliated (chain) clinic/veterinarian	8	4	18	16	18
Mobile pop-up clinic/veterinarian	6	5	6	3	5
Cat only clinic					
Clinic/veterinarian affiliated with veterinary college	1	4	2	2	1
Other	2	0	0	0	0
**Cats**	n = 73	n = 71	n = 67	n = 41	n = 80
Local independent clinic/veterinarian	71	82	67	71	63
Nationally affiliated (chain) clinic/veterinarian	12	10	22	15	18
Mobile pop-up clinic/veterinarian	5	4	6	5	10
Cat only clinic	5	3	0	2	9
Clinic/veterinarian affiliated with veterinary college	3	1	4	5	1
Other	3	0	0	2	0

**Table A6:** Affiliation of veterinary clinic respondent most often frequents, by income.

Affiliation	$0–$24,999	$25,000–$49,999	$50,000–$74,999	$75,000–$99,999	$100,000 and Higher
**Dogs**	n = 84	n = 106	n = 83	n = 63	n = 144
Banfield Pet Hospitals^®^	7	7	12	21	22
Veterinary Centers of America (VCA)	18	11	24	16	23
BluePearl	6	2	7	8	12
Pet Partners	7	5	5	14	9
None of the above	30	38	36	44	35
I don’t know	40	42	24	16	24
**Cats**	n = 73	n = 71	n = 67	n = 41	n = 80
Banfield Pet Hospitals^®^	8	10	15	32	40
Veterinary Centers of America (VCA)	21	17	30	24	41
BluePearl	7	3	9	12	21
Pet Partners	8	7	6	22	16
None of the above	34	56	45	68	64
I don’t know	47	62	30	24	44

**Table A7:** Veterinary care spending by income categories (%).

Spending	$0–$24,999		$25,000–$49,999		$50,000–$74,999		$75,000–$99,999		$100,000 and Higher	
	Dogs	Cats	Dogs	Cats	Dogs	Cats	Dogs	Cats	Dogs	Cats
$0	14	31	12	24	11	19	10	27	3	16
$0.01–$75	39	32	17	16	22	35	22	33	22	32
$76–$150	23	20	31	30	35	23	33	19	26	23
$151–$225	12	8	24	16	13	12	9	8	15	12
$226–$300	3	4	5	5	9	3	7	6	7	6
$301–$375	2	1	4	5	1	6	4	4	5	0
$376–$450	2	1	0	1	1	1	0	0	5	6
$451–$525	0	0	0	1	2	0	6	2	7	1
$526–$600	1	0	3	0	0	0	3	0	1	1
$601–$675	0	0	1	0	1	0	0	0	1	0
$676–$750	1	0	0	1	0	1	1	0	2	0
$751–$825	1	0	1	0	0	0	1	0	0	0
$826–$900	0	1	0	0	0	0	1	0	1	1
$901–$975	0	0	0	0	0	0	0	0	1	0
$976+	0	1	3	0	5	0	1	2	3	1

**Table A8:** Dog owner spending on veterinary related items, percentage of dog (n = 480) owners, by income (%).

Spending	Low-Cost Spay/Neuter Clinic	Low-Cost Vaccination Clinic	Veterinarian/Clinic/Practice of any kind	Emergency Veterinary Clinic	Ambulatory Veterinary Services ^1^	Veterinary College Services	Veterinary Surgery Center	Specialty Veterinary Center/Clinic ^2^	Other
$0	10	9	6	7	3	5	4	0	53
$0.01–$75	28	29	19	21	45	37	29	22	0
$76–$150	32	37	29	22	24	32	22	22	20
$151–$225	15	14	19	13	3	11	16	15	7
$226–$300	4	4	8	8	14	5	7	11	0
$301–$375	3	3	5	4	0	5	7	7	0
$376–$450	2	1	3	7	7	0	2	7	0
$451–$525	0	1	4	7	0	0	7	7	7
$526–$600	2	1	1	1	0	0	2	0	7
$601–$675	1	0	1	1	0	0	2	0	0
$676–$750	0	0	1	3	3	0	0	0	0
$751–$825	0	0	1	0	0	0	0	0	0
$826–$900	1	0	0	1	0	0	0	0	7
$901–$975	1	1	0	0	0	0	0	0	0
$976+	1	1	3	6	0	5	2	7	0

^1^: i.e., in-home care; ^2^: e.g., allergy testing, ophthalmologist, etc.

**Table A9:** Cat owner spending on veterinary related items, percentage of cat (n = 332) owners, by income (%).

Spending	Low-Cost spay/Neuter Clinic	Low-Cost Vaccination Clinic	Veterinarian/Clinic/Practice of any Kind	Emergency Veterinary Clinic	Ambulatory Veterinary Services ^1^	Veterinary College Provided Services	Veterinary Surgery Center	Specialty Veterinary Service Center/Clinic ^2^	Other
$0	25	14	14	9	0	13	0	0	87
$0.01–$75	29	42	23	25	47	56	56	38	0
$76–$150	19	23	32	27	12	13	13	23	4
$151–$225	9	8	15	20	24	6	13	23	4
$226–$300	6	4	7	7	12	13	6	8	0
$301–$375	5	4	4	5	0	0	6	8	0
$376–$450	3	1	1	2	6	0	6	0	4
$451–$525	1	1	1	0	0	0	0	0	0
$526–$600	0	0	0	0	0	0	0	0	0
$601–$675	0	0	0	0	0	0	0	0	0
$676–$750	1	1	0	0	0	0	0	0	0
$751–$825	0	0	0	0	0	0	0	0	0
$826–$900	1	1	1	2	0	0	0	0	0
$901–$975	0	0	0	0	0	0	0	0	0
$976+	1	2	1	4	0	0	0	0	0

^1^: i.e., in-home care; ^2^: e.g., allergy testing, ophthalmologist, etc.
